# A novel continuous inhibitory-control task: variation in individual performance by young pheasants (*Phasianus colchicus*)

**DOI:** 10.1007/s10071-017-1120-8

**Published:** 2017-08-09

**Authors:** Christina Meier, Sara Raj Pant, Jayden O. van Horik, Philippa R. Laker, Ellis J. G. Langley, Mark A. Whiteside, Frederick Verbruggen, Joah R. Madden

**Affiliations:** 1 School of Psychology, University of Exeter, Washington Singer Laboratories, Exeter, EX4 4QG UK; 2 Experimental Psychology, Ghent University, Ghent, Belgium

**Keywords:** Pheasant, *Phasianus colchicus*, Inhibitory control, Stop-signal task, Detour-reach task, A-not-B task

## Abstract

Inhibitory control enables subjects to quickly react to unexpectedly changing external demands. We assessed the ability of young (8 weeks old) pheasants *Phasianus colchicus* to exert inhibitory control in a novel response-inhibition task that required subjects to adjust their movement in space in pursuit of a reward across changing target locations. The difference in latencies between trials in which the target location did and did not change, the distance travelled towards the initially indicated location after a change occurred, and the change-signal reaction time provided a consistent measure that could be indicative of a pheasant’s inhibitory control. Between individuals, there was a great variability in these measures; these differences were not correlated with motivation either to access the reward or participate in the test. However, individuals that were slower to reach rewards in trials when the target did not change exhibited evidence of stronger inhibitory control, as did males and small individuals. This novel test paradigm offers a potential assay of inhibitory control that utilises a natural feature of an animal’s behavioural repertoire, likely common to a wide range of species, specifically their ability to rapidly alter their trajectory when reward locations switch.

**Electronic supplementary material:**

The online version of this article (doi:10.1007/s10071-017-1120-8) contains supplementary material, which is available to authorized users.

## Introduction

Flexibility in an individual’s behaviour is most evident when subjects quickly adapt their behaviour to unexpectedly changing external demands, for example by inhibiting inappropriate or no longer relevant behaviour, or adjusting an action that has already been initiated (Jurado and Rosselli 2007; Ardila 2008; Chan et al. 2008; Suchy 2009). Such flexibility is considered to be a product of the inhibitory control exerted by that individual and is commonly deemed to indicate an aspect of their cognitive performance (Coutlee and Huettel 2012).

Cognitive flexibility in terms of inhibitory control is reported in a wide range of animal species and has been assayed using tasks such as detour-reach tasks (e.g. song sparrows *Melospiza melodia,* Boogert et al. 2011; dogs *Canis lupus familiaris,* Bray et al. 2015; 36 species, MacLean et al. 2014; Clark’s nutcrackers *Nucifraga columbiana,* Vernouillet et al. 2016), A-not-B tasks (e.g. dogs, Bray et al. 2014; New Caledonian crows *Corvus moneduloides,* Jelbert et al. 2016; MacLean et al. 2014; equids, Osthaus et al. 2013) and reversal-learning tasks (e.g. Capuchin monkeys *Cebus apella,* Beran et al. 2008; Boogert et al. 2011; rats *Rattus norvegicus,* Floresco et al. 2008; North Island robins *Petroica longipes,* Shaw et al. 2015). There is some debate as to exactly what aspects of cognitive flexibility each of these tests reveals and how well they correlated may be one to another (Audet and Lefebvre 2017). The human literature on inhibitory control offers a range of additional psychological paradigms that may be adopted to assess inhibitory control in non-human animals. One widely used paradigm to assess inhibitory control in terms of behavioural inhibition is the stop-signal task (Logan and Cowan 1984; for a review, see Verbruggen and Logan 2008), in which subjects complete a series of trials by quickly responding (often under time constraint) to a presented stimulus (the “Go stimulus”). Occasionally, the Go stimulus is followed by an additional signal; on such signal trials, the subject should withhold the response that would have been required if no signal had occurred. In a “stop-change” variant of this paradigm, subjects may also have to replace the initially required response with a different one (Verbruggen and Logan 2009; Boecker et al. 2013). Computerised versions of stop-signal and stop-change tasks have been used successfully not only with humans but also with macaques *Macaca mulatta* (i.e. Emeric et al. 2007; Liu et al. 2009; Stuphorn et al. 2010), baboons *Papio papio* (Lacreuse et al. 2016) and rats (i.e. Bari et al. 2011; Beuk et al. 2014; Eagle and Robbins 2003a, b; Mayse et al. 2014). Evidence from behavioural, neuropsychological and computational studies suggests that successful performances in stop-signal and stop-change tasks require capacities for inhibitory control (i.e. Chan et al. 2008; Jurado and Rosselli 2007; Lipszyc and Schachar 2010; Salthouse 2005; Suchy 2009). More specifically, being able to stop or change a behaviour after it has been initiated necessitates a chain of cognitive processes, including detecting the relevant signal, selecting the appropriate action (e.g. cancelling a response, or selecting an alternative response), and implementing the selected action (Verbruggen et al. 2014; Verbruggen and Logan 2015).

In addition to discrete versions of the stop-signal tasks, some studies have used continuous variants. For example, Verbruggen and McLaren (2017) used a continuous version of a stop-signal task to assess the development of inhibitory control across the early lifespan (ages 4–11 and 18–21) of humans. In their computer-based paradigm, participants had to quickly move a mouse cursor towards a target presented in a specific location on the computer screen; on a minority of trials, the target moved to a different location after the subjects started moving the mouse cursor towards it, forcing them to stop and change their mouse movements. Verbruggen and McLaren (2017) found that older children and adolescents, who possess a more mature level of inhibitory control, could quickly change the movement of the mouse cursor (i.e. they were able to quickly stop moving to the old target location and instead start moving to the novel location on trials in which the change was required). Conversely, younger children, whose ability to exert inhibitory control was still limited, were slower to adjust the mouse trajectory. Procedurally, this paradigm is very similar to double-step tasks (Georgopoulos et al. 1981; Pélisson et al. 1986; Oostwoud Wijdenes et al. 2016), in which subjects start making a movement (of their hand, a mouse cursor, a saccade, etc.) towards a target, which sometimes changes its location before the subject has reached it. Like stop-signal tasks, double-step tasks are assumed to involve inhibitory-control processes (Ray et al. 2004; Wilimzig et al. 2010; Thakkar et al. 2015) and have been used in non-human primates (Umeno and Goldberg 1997; Camalier et al. 2007). The task does not require any extensive training to acquaint subjects with the task requirements; it therefore reduces the influence of learned contextual responses on performance. Unlike paradigms such as detour-reach, A-not-B or reversal-learning tasks, there is clear evidence that stop-change paradigms such as Verbruggen and McLaren’s afford inhibitory-control processes that enable subjects to stop one action and change to another one (Verbruggen and Logan 2009; Boecker et al. 2013). Individual differences in performance may therefore relate more directly to differences in inhibitory control (broadly defined; as mentioned above, inhibitory control involves a chain of processes). Specifically, subjects that possess efficient inhibitory control would be expected, after initially approaching the location at which the target was presented at the start of a trial, to alter direction towards the new location soon after the correct location changes, and before the old (now incorrect) location is reached. Conversely, subjects that lack control might continue towards (and reach) the original target location even after it is signalled that the original location is no longer correct.

Although research on inhibitory control in animals has mainly investigated differences between species, with the implied assumption that individuals of a species or population will perform similarly (MacLean et al. 2014; Beran 2015; Jelbert et al. 2016), it is likely individuals within the same species vary in their ability to exert inhibitory control (cf. Shaw 2016). In healthy human adults, there is great variation in the ability to control the initiation of behaviour, partly due to hereditary influences (Friedman et al. 2008; Miyake and Friedman 2012). Such differences may manifest in personality traits of high or low impulsivity (cf. Sharma et al. 2014). Emotional and motivational factors may also contribute to individual differences in inhibitory control (Pessoa 2009; Botvinick and Braver 2015); for example, Padmala and Pessoa (2010) showed that responses that had previously been rewarded were harder to suppress than previously unrewarded responses.

In non-human animals, differences in inhibitory control have been considered comparatively across species. Inter-species differences in inhibitory control may be concealed or confounded by non-cognitive factors. Differences in morphology and metabolism might account for higher or lower levels of inhibitory control amongst species (Speakman 2005; MacLean et al. 2014; Beran 2015). In particular, species with greater body mass may be able to exert more inhibitory control than species with lower body mass. This finding has been linked to metabolic rate: species with fast metabolic rates presumably have to consume more energy to preserve their body weight and may thus be less able to forego a potential food source than species with slower metabolic rates (Speakman 2005; MacLean et al. 2014; Beran 2015). A similar positive relationship between body size or metabolic rate and the exhibition of inhibitory control may be seen for individuals of the same species. Alternatively, on a task involving inhibition of physical movement such as we deploy, larger individuals may be less able to exhibit inhibition because of greater momentum.

We investigated the ability of pheasants *Phasianus colchicus* to exert inhibitory control when adjusting their movement in space in pursuit of a reward across changing target locations. Specifically, we explored inter-individual differences in inhibitory control, and whether test performance could be explained by non-cognitive measures such as an individual’s morphology or reward motivation. Pheasants participate in a range of cognitive tasks including assays of problem-solving (van Horik and Madden 2016), motor skills (Whiteside et al. 2015), spatial memory (Whiteside et al. 2016) and discrimination and association tasks (van Horik et al. 2017). Inter-individual differences in performance in these tasks may be explained by different early rearing environments such as diet or habitat complexity and can have fitness consequences in terms of adult mortality rates (Whiteside et al. 2015, 2016). Young pheasants are precocial at hatching and so can be reared in large numbers, under controlled conditions in the absence of adults. This permits a high degree of standardisation in their early-life conditions (or robustly controlled experimental variation), which reduces the effects of differential experiences early in life confounding responses to testing. Despite a standardised rearing environment, individual differences in participation in tasks may be explained by an individual’s sex and body condition (van Horik et al. 2017). Therefore, we considered how these non-cognitive factors may influence performance in the current test.

## Methods

### Subjects

Two hundred pheasant chicks were hatched from an incubator on the same day in May 2015 and randomly allocated to one of four replicated enclosures, with 50 birds per enclosure at North Wyke Farm, Devon, UK. For the first 2 weeks, birds were housed in an indoor heated shed within 2 m × 2 m enclosures. At 2 weeks, birds could access an unheated, grass floored run of 1.5 m × 4 m. At 3 weeks old, birds could access an outdoor enclosure of 4 m × 8 m, which was roofed with mesh. Commercially supplied, age-specific food (Keeper’s Choice starter and grower feeds) and water were provided ad lib, and each enclosure was supplied with the same perching and shelter opportunities. At this time, all birds were affixed numbered plastic patagial wing tags.

### Apparatus

**Fig. 1:**
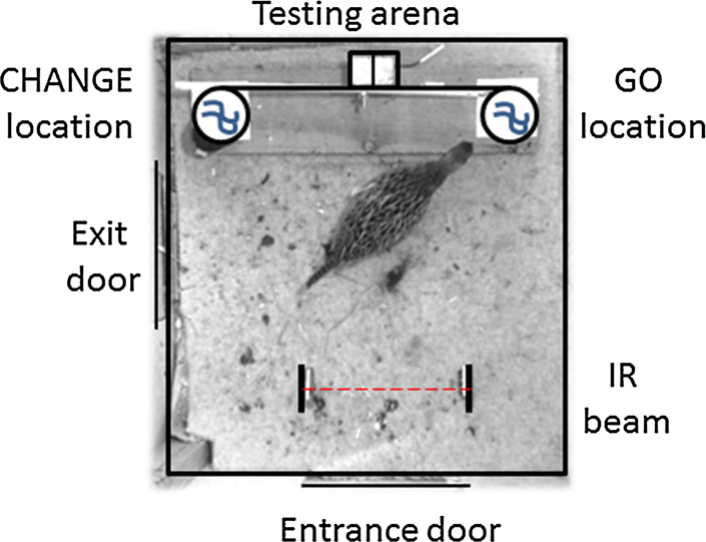
Experimental apparatus used to test inhibitory control in pheasants (not to scale, see text for details on dimensions). The *dotted red line* indicates the infrared beam that was crossed as the pheasant moved towards the Go or Change locations. Each location was baited with two mealworms, illustrated as *blue curved lines*. The Change location was covered by a black cylinder on Go trial; on Change trials, there was an additional black cylinder attached to the see-saw (*black horizontal line*) that covered the Go location once the pheasant crossed the infrared beam, whilst the Change location was uncovered. For enclosures A and C, the Change location and exit door were on the *left-hand side* as depicted; for enclosures B and D, the set-up was mirrored, with the Change location and exit door on the *right-hand side* (colour figure online)

 Pheasants aged 8 weeks entered a 75 cm × 75 cm testing arena (Fig. 1) via a sliding door (25 cm × 25 cm) that separated the arena from the pretest enclosure. A second hinged door (25 cm × 25 cm) was situated in the lower middle of one of the walls allowing an exit to a post-test recovery area. This exit was located to the left of the entrance for the arenas accessible from enclosures A and C and to the right of the entrance for the arenas accessible from enclosures B and D. The four arenas were in visual but not auditory isolation from other birds.

An apparatus was mounted along the wall opposite to the arena entrance and consisted of a white plastic beam of 60 cm that was hinged on a bolt attached to a block of wood at 10 cm off the ground. The block of wood was mounted onto a board of wood 60 cm × 15 cm × 2 cm. A 10 cm × 10 cm white Perspex baseplate was screwed flat onto each end of the board to indicate the two food locations, the centres of which were 50 cm apart from each other (see Fig. 1). Two opaque cylinders made from black paper could be attached to the two ends of the see-saw beam to cover the food location. The beam rested on the armature of a solenoid integrated into the wooden block that held the see-saw hinge. The armature retracted when the solenoid was activated, allowing the see-saw to tip over.

Approximately 15 cm from the right side of the arena entrance into the arena, an IR-LED was mounted 7 cm from the ground, which continuously generated an infrared beam. The beam was detected by an infrared sensitive lux meter mounted at the same height 15 cm into the arena from the left side of the entrance. The lux meter recorded any changes in lux of the incoming infrared beam and submitted this information to an Arduino One microchip board; if there was an interruption in the light beam, the Arduino One board operated the solenoid attached to the see-saw. A Kenvo HDV-601S video camera was attached directly above each arena to film the trajectories of the participating birds.

### Procedure

From 1 day old, the pheasants were habituated to experimenters, the arena and being tested in isolation. They were trained to enter the arena through the entrance door of their own volition when an audible cue sounded (see van Horik et al. 2017). When pheasants were 8 weeks old (after the time when they would naturally be independent of their mother (Hill and Robertson 1988), they all received a series of habituation sessions in which the see-saw was fixed in place and the two food locations were exposed, both baited with two mealworms, which had been freshly killed to prevent their movement. Both locations were visible from the entrance door to the testing arena. The pheasants were allowed to freely explore the testing arena, in isolation, and feed from the two food locations. A pheasant received up to three habituation sessions (maximum duration of 5 min) until it fed from both food locations in the same session. During the habituation sessions, the see-saw beam was moved manually to familiarise the birds with its motion.

Test trials were administered in blocks of two trials a day (09:00–13:00 and 14:00–18:00), on three consecutive days. Trials 1, 2, 4 and 5 were ‘Go’ trials, in which the food location closest to the arena exit (henceforth called the Change location—see Fig. 1) was covered by an opaque cylinder; the opposite food location (henceforth referred to as the Go location) was exposed and baited with two mealworms (~20 mm length each), visible from the arena entrance. The opaque cover was not attached to the see-saw, so that, when the pheasant crossed the infrared light beam and thus caused the see-saw to tip over, the cover was not lifted. The Go location remained available, and the pheasant was allowed to feed freely from that location. Trials 3 and 6 were ‘Change’ trials: in these trials, two opaque cylinders were attached to the two ends of the see-saw beam, and both food locations were baited with two mealworms. Initially, the see-saw was positioned as in Go trials, so that the Change location was covered by the opaque cylinder and the Go location was exposed. When the pheasant crossed the infrared light beam, the Change location became exposed, whilst the Go location became covered by the opaque cylinder on the other end of the see-saw. In these trials, the pheasant was allowed to feed from the newly exposed Change location. A trial ended once the pheasant had consumed all the food from the available food location; at this point the exit door was manually opened and the bird was allowed to leave to the post-test recovery area (see ESM Videos 1 and 2).

### Measures of morphology and motivation

At 10 weeks old, 1 week after the testing was completed, all birds were caught and their mass was recorded using Samson scales (precision 5 g). To account for individual differences in stride length, we also measured the pheasants’ tarsus length using callipers (precision 0.1 mm). To reflect an individual’s overall body size, a principal component was extracted that explained 91% of the variation in a subject’s body mass and tarsus length.

We collected two measures of their motivation to interact with the test set-up. These are detailed in van Horik et al. (2017), along with confirmation of their repeatability over time and across contexts. In brief: Our first measure (Test Order, TO) considered the order in which each individual left the communal pretest enclosure of their own volition and entered the testing arena in 37 trial sessions for a range of other psychometric tasks unrelated to the current study (including tests of spatial memory, colour and shape discriminations, reversals and motor skills—none of which utilised the see-saw apparatus). Individuals that were amongst the first to enter the test arena had a low TO score. Because subjects entered the testing arena freely, TO represented a measure of their willingness to enter the testing arena. This could comprise two elements: First, an enthusiasm to participate in a test may encourage subjects to enter the test arena. Second, subjects may wish to escape the denser social environment of the enclosure. It remains difficult to separate these two influences or disentangle any interaction in their effects. TO could also indicate the time since a bird’s last access to food, with higher TO scores indicating a longer delay since their last opportunity to feed. However, this cannot reliably indicate time since last feed because the bird may not have eaten immediately prior to the removal of food. Our second measure (Baseline Worm Acquisition, BWA) was based on each individual’s latency to acquire the freely available mealworm that was positioned on top of the test apparatus used in each of these previous 37 unrelated psychometric tasks. Individuals that failed to acquire the baseline worm were allocated a ceiling value of 120 s. This measure provided us with an indication of their willingness to approach the test apparatus, but may also indicate something of the bird’s hunger and hence food motivation. Low BWA scores indicate that the bird took the baseline worm quickly upon entering the testing arena.

### Measures of inhibitory control

We described an individual’s level of inhibitory control using three dependent variables. As our first measure, we calculated the difference in the latencies to complete the last Go trial (trial 5) and the last Change trial (trial 6). In humans, reaction times on successful trials have been found to be longer in trials in which inhibition is required (i.e. Change trials) compared to trials in which no inhibition is required (i.e. Go trials, Verbruggen and Logan 2009).

Additionally, we determined the subjects’ trajectory paths as they moved from the entrance door to the correct food location. Using the Open Source Physics Tracker© video tracking software (Brown 2009), the coordinates of a pheasant’s centre of mass inside the arena were extracted from the videos of each trial. The axes of coordinates were standardised so that the point of crossing the infrared beam coincided with the point of crossing the *x*-axis at (*x*, 0); the available food location in Go trials was located at (1, 1) and the available food location in Change trials was located at (−1, 1). To account for individual differences in the latencies to reach the correct food location, the trajectory paths of each trial were standardised in a way similar to Vincentization (Vincent 1912; Ratcliff 1979; Genest 1992; Rouder and Speckman 2004); that is, the value of the latency for a given trial was split into twenty 0.05-quantile points; the coordinates at each quantile point were connected to create the trajectory path for that trial. These were then averaged across individuals to calculate the mean trajectory paths for each of the six trials.

**Fig. 2:**
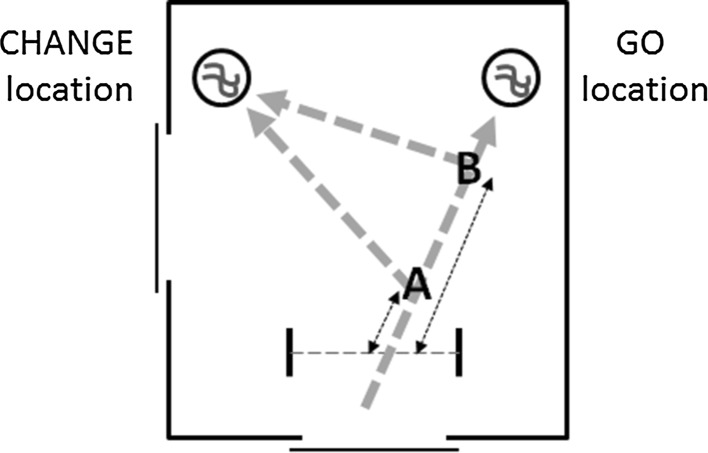
An individual’s trajectory-correction point on trial 6 was determined as the point at which a pheasant started moving distinctly towards the Change location. The measures for analysis were both the distance the pheasant had moved since crossing the infrared beam (indicated by the *double-headed arrows*)—for example, the shorter distance to Point *A* indicates an individual exerting stronger inhibitory control compared to an individual not deviating until Point *B*—and the time taken to cover this distance (the change-signal reaction time)

From the trajectory data, we determined an individual’s trajectory-correction point in trial 6, which was the last Change trial (see Fig. 2). The trajectory-correction point is defined as the inflection point in a pheasant’s trajectory path, at which the *x*-coordinate of the trajectory reached its maximum positive value. It indicates the moment at which the subjects, after initially approaching the (incorrect) Go location (with a positive *x*-coordinate), started approaching the (correct) Change location (with a negative *x*-coordinate). We considered both spatial and temporal characteristics of this correction point as our second and third measures of inhibitory control. For our second measure, we calculated the distance of the correction point from the point at which the infrared beam was crossed and the change signal occurred (henceforth referred to as the IR crossing point). The relative distance indicates how quickly a subject altered its behaviour in response to the change in target location: shorter distances indicate efficient behavioural adjustment and inhibitory control, whilst larger distances indicate slower adjustment and presumably less control over behaviour (see Fig. 2). For our final measure, we recorded the time taken to cover the distance between IR crossing point and trajectory-correction point (henceforth referred to as the change-signal reaction time). The change-signal reaction time is a direct measure of the time afforded by inhibitory-control processes; it relates to the stop-signal reaction time that is estimated across several trials in conventional stop-signal paradigms (see Verbruggen and Logan 2008, for a discussion of stop-signal reaction times).

In order to summarise these three measures and generate a single measure of an individual’s inhibitory control, we performed a principal component analysis on the three measures and extracted a single component that we could use in subsequent analyses. The score on this component was multiplied by −1, so that birds with a low score possessed low inhibitory control. This summary measure meant that we could avoid problems of multiple testing using three likely related measures of the same putative process; inhibitory control.

### Data exclusion criteria

Of the 194 pheasants that took part in this experiment (six pheasants had died before this experiment commenced), 113 subjects failed to complete the task in less than 2 min in at least one of their six trials; their data were excluded from analyses. This exclusion criterion was applied for two reasons. First, birds that had not accessed visible food within 2 min were likely to be either unmotivated by food and/or more motivated by a desire to leave the testing chamber perhaps because of stress. This was supported by our observation in similar test paradigms that if the reward was not eaten within 2 min, it was commonly not eaten at all, with the bird becoming increasingly anxious and keen to leave the testing chamber. Second, if birds had not accessed the food within 2 min, then their movement pattern was typically very tortuous making accurate calculation of a change point unreliable. Further, to ensure that our measures captured the effects of inhibiting a response that had already been initiated, we only included the data of subjects that had, on trial 6, pursued a direct path towards the Go location when that location had still been indicated as the correct location (that is, before the pheasant reached the IR crossing point, at which the location changed). Subjects whose path from the entrance door to the IR crossing point deviated from a straight line to the Go location by more than 33% of the length of that line were excluded; this affected 14 subjects. Due to a technical malfunction, full sets of measures were only available for three of the four enclosures (leading to the exclusion from analysis of a further eleven birds), which left 56 birds for which there was a complete data set available to analyse.

### Statistical analysis

One main concern for statistical analyses was to determine the trial in which inhibition—or a lack thereof—would affect performance most evidently. As stated above, we assumed that inhibitory control would be required to perform adequately in Change trials, resulting in longer reaction times in Change trials compared to Go trials. However, this might not be the case for trial 3, in which the pheasants were exposed to a change for the first time: as they had no experience with any change in response requirements upon commencing this trial, it seems unlikely that the pheasants would have been able to respond to the change signal and adjusting their behaviour before reaching the Go location. Instead, it seems logical to assume that the pheasants continued towards the Go location despite it being covered and searched for alternate food sources only after encountering that the Go location was inaccessible. Conversely, having made this experience on trial 3 might consequently have enabled the pheasants to adjust their behaviour more efficiently, guided by inhibitory control, in trial 6.

To test whether response times were longer on Change trials than on Go trials, we performed a repeated-measures ANOVA on the times taken to complete each trial (i.e. the response times from entering the arena to reaching the correct food location), including both Block (first 3 trials, last 3 trials) and Trial Type (first Go trial, second Go trial, Change trial) as within-subjects factors. Furthermore, we assumed that response times on the second Go trial in each block should be shortest within that block, as subjects may show a performance benefit from being able to repeat the response that was reinforced in the first Go trial in the second Go trial. This assumption was tested by assessing any quadratic trend observed in the response times of the three trials in each block.

To assess whether inhibitory control was influenced by sex, morphology or motivation, we calculated a generalised linear mixed model (GLMM) using a normal distribution and inhibitory-control scores as the target variable. Fixed effects included an individual’s sex and body size, and Baseline Worm Acquisition and Test Order scores as measures of reward motivation. To account for sexual size dimorphism, which was already apparent at the time of testing, the two-way interaction between sex and body size was included as a fixed effect in the model. To control for an individual’s overall speed, the latency to complete trial 4 was included as a further fixed effect. Enclosure was included as a random effect. Descriptive statistics of the inter-individual range in sex, morphology, motivation, response times in trial 4 and inhibitory control scores are presented in Table 1.

**Table 1:** Descriptive statistics of the inter-individual range, mean and standard deviation in the sex, body size, Baseline Worm Acquisition (BWA) rate, Test Order (TO), the time taken to complete trial 4 and inhibitory control (IC) score of 56 pheasants tested

	Sex	Body size	BWA	TO	Trial 4 RT	IC score
Range	22F, 34M	5.43	46.4	33	24.2	4.64
Min	–	−2.62	1.4	8	1.1	−3.09
Max	–	2.81	47.8	41	25.3	1.55
Mean	–	0.12	4.2	22	5.0	−0.09
SD	–	1.01	6.5	8	4.9	1.05

### Ethical note

All work was approved by the University of Exeter Psychology Ethics Committee and conducted under Home Office licence PPL30/3204. The raw data are available at http://hdl.handle.net/10871/28542. Original video recordings are available from JRM. Birds were habituated to human observation from 1 day old. Shaping procedures, using meal-worm rewards, were adopted to habituate subjects to the testing arena. These procedures were considered to mitigate stress and encouraged subjects’ voluntarily participation during testing. Birds could therefore choose whether or not to participate in tasks. There were no enforced aversive stimuli. In order to encourage participation in the tests, birds were removed from their normal food supply (but not water) for up to 2 h before testing whilst in the holding section. Birds were reared at a lower density than that recommended by DEFRA’s code of practice, thus reducing likely stress and competition between chicks.

## Results

### Which trials require inhibition?

**Fig. 3:**
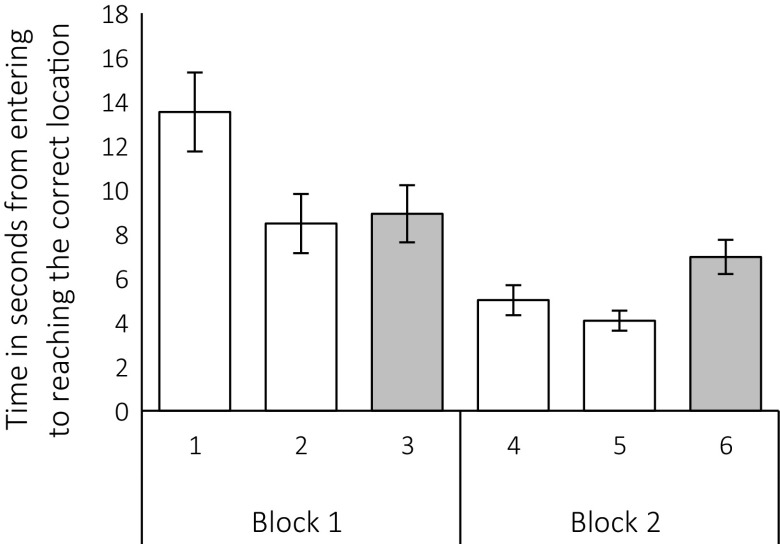
Latencies from entering the arena to reaching the correct food location for trials 1–6, split into blocks *1* and *2*. *Error bars* represent standard errors. *Note* trials 3 and 6 (*shaded bars*) were Change trials; the remaining trials were Go trials

Overall, Change trials were completed more slowly than Go trials (main effect of Trial Type: *F*(2, 106) = 3.63, *p* = .037, Fig. 3). Furthermore, response times were slower in the first block of three trials compared to the last block of trials (Block: *F*(1, 53) = 32.61, *p* < .001), and there was a significant interaction between the two factors [*F*(2, 106) = 6.99, *p* = .003]. Bonferroni-corrected post hoc comparisons revealed that in block 1, pheasants took longer to reach the correct location in their first Go trial (*M* = 13.3 s, SD = 13.1 s) compared to the second Go trial (*M* = 8.5 s, SD = 9.7 s), *p* = .016. Comparisons of trial 1 to the Change trial 3 (*M* = 8.8 s, SD = 9.4 s) and of trial 2 to trial 3 each did not show any significant differences, both *p* ≥ .24. A significant quadratic trend confirms that the pheasants became faster from trial 1 to subsequent trials in block 1 [*F*(1, 54) = 5.05, *p* = .029]. For block 2, the time taken to complete trial 6 (*M* = 6.9 s, SD = 5.7 s) was significantly longer than the latency to complete trial 5 (*M* = 4.1 s, SD = 3.3 s), *p* = .006. The two remaining pairwise comparisons between trials 4 (*M* = 5.0 s, SD = 4.9 s), 5 and 6 did not show any significant differences, both *p* ≥ .21. This observation is confirmed by a significant quadratic trend across trials in block 2 [*F*(1, 54) = 10.87, *p* = .002]. Critically, the above results indicate that performance on trial 3, in which the pheasants incurred a change in target location for the first time, might be too variable to accurately investigate the effects of inhibitory-control processes.

**Fig. 4:**
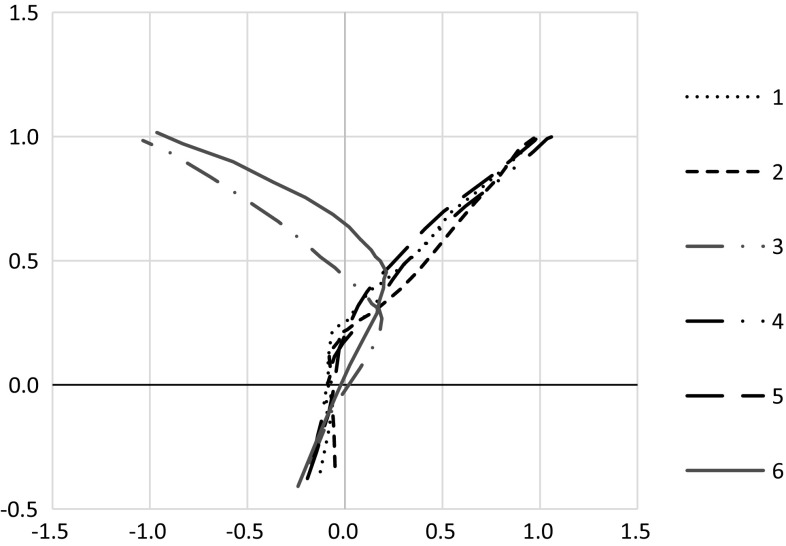
Average trajectory paths for trials 1–6. *Note* the Go location, which was rewarded in trials 1, 2, 4 and 5, is at (1, 1), and the Change location, which was rewarded in trials 3 and 6, is at (−1, 1). On those trials, the location changed from Go to Change when the pheasant crossed the IR beam at *y* = 0 (*black horizontal line*)

Another indicator that the first block of trials might not provide an adequate measure of inhibitory control is shown in Fig. 4, which shows the trajectory paths of each trial. In trial 3, the pheasants were closer to the IR crossing point when correcting their path towards the changed food location than they were in trial 6 (trajectory-correction point on trial 3: *M* = 0.57, SD = 0.57; trial 6: *M* = 0.69, SD = 0.60). Yet their average change-signal reaction time was slower in trial 3 (*M* = 3.3 s, SD = 7.66) than in trial 6 (*M* = 2.7 s, SD = 4.0); however, note that neither of these comparisons are significantly different, both *p* ≥ .15.

Nonetheless, to account for the possibility that no inhibitory control was employed to solve the first Change trial, we concentrated our subsequent analyses on the second block of trials, especially the differences between trial 5, the final Go trial, and trial 6, the final Change trial.

### Is pheasant behaviour indicative of inhibitory control?

The differences in latencies between trials 5 and 6 were not because the birds were simply closer to the Go location than to the Change location when it was revealed. The trajectory paths (Fig. 4) indicate that in all trials the pheasants were close to the centre of the arena at the IR crossing point, as indicated by the *x*-coordinate being close to zero at *y* = 0 (test of *x*-coordinate against zero in trial 6: *M* = 0.01, SD = 0.18, *t*(55) = 0.26, *p* = .79). Pheasants crossed the infrared beam at the same point in all trials of block 2, indicated by the *x*-coordinate being comparable in trials 4–6 [*F*(2, 110) = 2.73, *p* = .07; mean *x*-coordinate on trial 4: 0.02, trial 5: 0.00, trial 6: −0.08, Fig. 4].

On trial 6, pheasants corrected their trajectory significantly after the change in target locations occurred, that is when they were further inside the test arena (indicated by the *y*-coordinate of the trajectory-correction point being significantly different from the IR crossing point at *y* = 0; for trial 6: *M* = 0.45, SD = 0.36, *t*(55) = 9.24, *p* < .001; Fig. 4). In addition, the trajectory-correction point, at which the bird switched from heading to the Go location to heading towards the Change location, was closer to the Go than to the Change location (indicated by the *x*-coordinate of the trajectory-correction point being significantly different from the middle of the arena at *x* = 0; for trial 6: *M* = 0.43, SD = 0.63, *t*(55) = 5.13, *p* < .001; Fig. 4). This indicates that the pheasants did not change their response immediately when the change occurred.

Birds typically completed the Go trial 5 faster than the Change trial 6, taking *M* = 2.8 s, SD = 6.3 s longer to reach the novel target. On trial 6, the distance between the IR crossing point and the trajectory-correction point ranged from 0 (correcting at the IR crossing point) to 2.18 (correcting after the Go location was surpassed), *M* = 0.70, SD = 0.60. The change-signal reaction time ranged from 0 to 21.5 s, *M* = 2.7 s, SD = 4.0 s. All three measures of performance correlated significantly with each other. The longer the time that an individual took to complete trial 6 compared to trial 5, the greater the distance from the IR crossing point to their trajectory-correction point (Pearson’s *R* = 0.58, *N* = 56, *p* < .003), and the longer their change-signal reaction time (*R* = 0.83, *N* = 56, *p* < .003). Similarly, the longer an individual’s change-signal reaction time, the longer the distance to their correction point (*R* = 0.44, *N* = 56, *p* = .003). The first component summarising these three measures had an eigenvalue of 2.26 and explained 75.24% of the variance in performance. Birds with a low score proceeded further towards the Go location, had longer change-signal reaction times and took increasingly longer to complete trial 6 compared to trial 5 and may be considered to exhibit weaker inhibitory control. Conversely, birds with a high score corrected their trajectory towards the Change location close to the IR crossing point, showed short change-signal reaction times and completed trial 6 at a same latency as, if not faster than, trial 5 and may be considered to exhibit stronger inhibitory control.

### Is inhibitory control predicted by morphology or motivation?

**Fig. 5:**
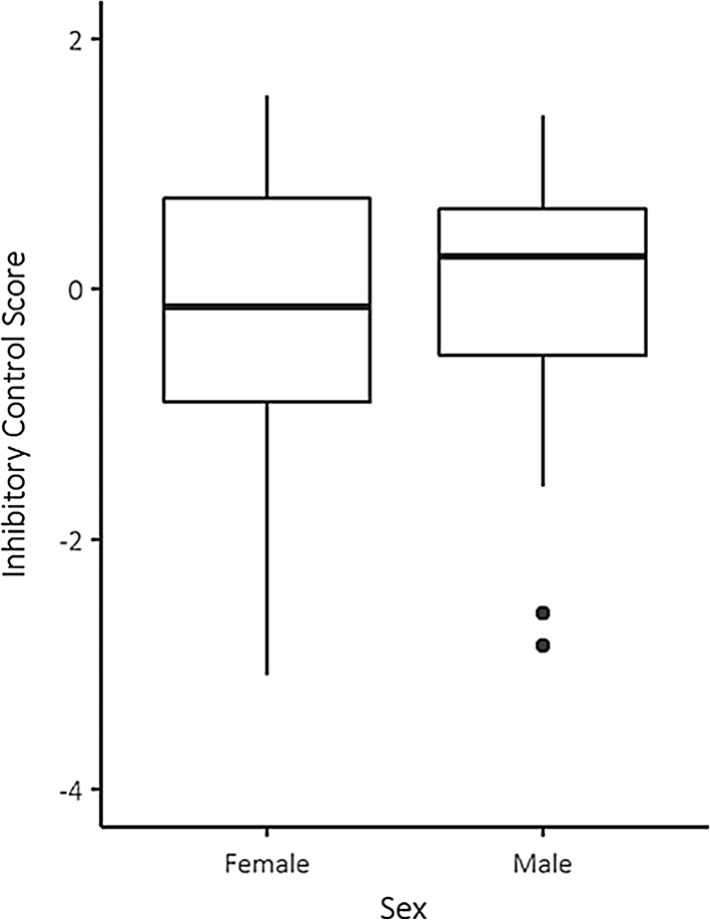
Inhibitory-control scores of male and female pheasants. High scores indicate strong inhibitory control

**Fig. 6:**
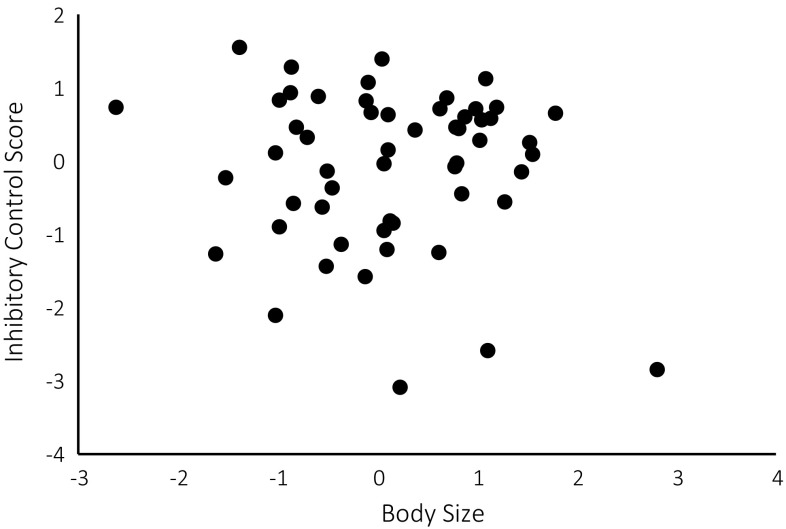
A weak, negative relationship between a pheasant’s body size (a composite score of its mass and tarsus length) and its inhibitory-control score. High scores indicate strong inhibitory control

**Fig. 7:**
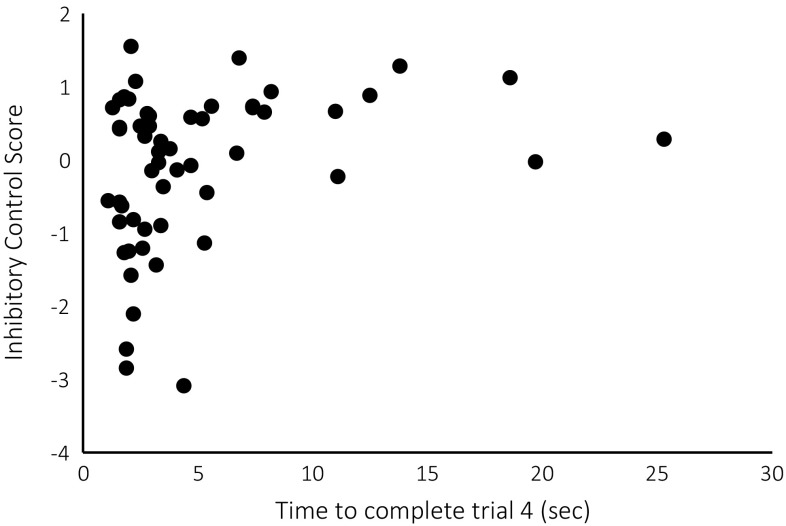
A weak positive relationship between the time that a pheasant took to complete the Go trial 4 and its inhibitory-control score

A pheasant’s score of inhibitory control was only marginally predicted by our measures of morphology or motivation [*F*(5, 49) = 2.20, *p* = .070]. As the interaction between sex and body size had no significant effect, it was removed and the analysis was repeated considering main effects only (Table 2). Males had a higher mean score on inhibitory control than females (males: *M* = −0.03, SD = 1.00; females: *M* = −0.20, SD = 1.15; *R* ^2^ = 0.01, *p* = .036, Fig. 5) as did birds with smaller body size compared to those with larger body size (*R* ^2^ = 0.01, *p* = .035, Fig. 6). Furthermore, pheasants that were slower to complete trial 4 showed better inhibitory control than faster pheasants (*R* ^2^ = 0.09, *p* = .037, Fig. 7).

**Table 2:** Coefficients, standard errors, *t* and *p* values of a generalised linear mixed model (GLMM) analysis on inhibitory-control scores as dependent variable

	Sex	Body size	BWA	TO	Trial 4 RT
Inhibitory control score					
*b*	−1.00	−0.48	0.02	0.00	0.07
SE	0.46	0.22	0.02	0.02	0.03
*t*	2.15	2.17	0.72	0.07	2.15
*p*	**.036**	**.035**	.47	.95	**.037**

*p* values < 0.05 are shown in bold

An individual pheasant’s sex and body size, Baseline Worm Acquisition (BWA) rate, Test Order (TO), and the time taken to complete trial 4 were included as fixed effects; enclosure was included as random effect

The reference value of sex was set to 0 (female)

## Discussion

### Do pheasants vary in their ability to exert inhibitory control?

We collected three measures of a pheasants’ inhibitory control in this continuous stop-change task: First, we measured the difference in latencies between the last Go and the last Change trials (trials 5 and 6). Pheasants took longer to reach the correct food location in the final Change-Signal trial compared to the previous Go trial. This suggests that inhibitory-control processes influenced performance on trial 6. Although longer response times on Change trials could potentially occur without the involvement in such processes, as subjects had to perform an altered response towards the changed target location on trial 6, the set-up of our arena prevented an increase in latencies. As the birds were forced to pass the IR crossing point in the centre of the arena, the distance to either food location was similar when the see-saw tipped over, and latencies to reach either location should be equally similar if subjects were able to initiate the altered response immediately without the need to inhibit the initial response. Second, we recorded the distance of the trajectory-correction point; some individuals moved to the Go location and even beyond it, whilst others pursued a trajectory towards the Change location from the moment of crossing the infrared light beam, when the see-saw was initiated. Third, we measured the change-signal reaction time on trial 6. For those birds that were able to correct their trajectory close to the IR crossing point, the change-signal reaction time was low, whilst others required several seconds to adjust their trajectory. These last two measures suggest that individuals differ in how rapidly they could adjust current trajectories. One explanation for why such differences occurred was that individuals differed in their inhibitory control performance.

Pheasants showed high intra-individual consistency in these three measures, reflected in the strong positive correlations between all three. An individual’s performance could be summarised by a single principal component; individuals with a low score were much slower to complete the Change trial than the previous Go trial, proceeded further towards the incorrect Go location and took more time before correcting their trajectory towards the correct Change location than individuals with a high score. The pheasants’ inter-individual differences resemble the performance of human children and adolescents in Verbruggen and McLaren’s (2017) task: young children, whose inhibitory-control skills are less developed, take longer to adjust their trajectories and to complete a Change trial than older children and adolescents. We believe that, as for Verbruggen McLaren’s task, performance in our paradigm can be considered indicative of inhibitory control.

### Can individual traits predict the ability to exert inhibitory control?

Performance in this task was predicted by sex and body size, but not by either measure of motivation. Curiously, the relationship we found with body size was in the opposite direction to that we predicted; across species, larger species may possess higher levels of inhibitory control than smaller species (Speakman 2005; MacLean et al. 2014; Beran 2015), and therefore, we might have assumed that within species, larger, better fed, individuals also exhibit more inhibitory control, provoking better performances, than smaller conspecifics (cf. Shaw 2016). However, smaller pheasants had better inhibitory-control scores than larger pheasants. Perhaps our measure of inhibitory control in larger individuals was confounded by their momentum in this physical task, with large individuals finding it harder to adjust their trajectory once the target changed. However, this does not explain why males, that in general are ~30% heavier than females, exhibited stronger inhibitory control. If body size per se was critical in determining performance in our task, we would have expected females to perform better than males. Males might be better at regulating their body movements than females and thus performed better in this task. The fact that those pheasants that took longer to complete trial 4 and thus were generally slower moving in the task even when no change had to be made, had higher inhibitory-control scores, might support this hypothesis.

We found no evidence that our assays of motivation to either escape a dense pretest holding area or enter a test arena baited with a food reward (as indicated by Test Order), or motivation to eat an accessible reward worm (as indicated by Baseline Worm Acquisition speed) explained variation in task performance. This contrasts to findings that human inhibitory control can be affected by reward motivation (Pessoa 2009; Padmala and Pessoa 2010; Botvinick and Braver 2015). Although motivation may reflect a readiness to engage with the task, it may not necessarily be related to sensitivity to task contingencies. Motivation or perseverance may lead to greater success in tasks that have a learning component simply because it increases the amount of contact with the test apparatus and reward contingencies (van Horik et al. 2017; van Horik and Madden 2016). When performance is unrelated to discovering a single optimal solution, high motivation alone may not be sufficient to explain inhibitory-control ability. High motivation may be found both in subjects with high inhibitory control and in subjects with low control; the former may be motivated to approach the rewarded food location, whereas the latter may be motivated to approach the food location that was rewarded most often. An individual’s performance may be simply a result of their reaction time, with generally faster birds exhibiting change earlier after having triggered the switch of the reward locations. We did not measure an individual’s reaction time in independent tests, so cannot exclude this.

### Is our task a good measure of inhibitory control?

Our novel paradigm allows the assessment of inhibitory control (broadly defined) over a behaviour that has been minimally trained, namely the ability to deviate from one trajectory when the location of a target changes, and can therefore be considered to be prevalent in the subjects’ natural repertoire. Our test subjects did not have to be trained to perform a different response on Change trials, as they corrected their behaviour on the first Change trial they encountered (although they did have to be familiarised with the test apparatus to overcome neophobia and learn that food rewards were accessible). Specifically, pheasants observed a target in the Go location and started to move towards it. When the location changed, the pheasants adjusted their approach to it, inhibiting their initial goal direction and changing to a new target. It might be possible to deploy the same paradigm across a wide range of disparate species, simply by resizing the testing field according to the size, visual field or movement speed of the study subject. This may require an alternative mechanism to conceal/reveal the rewards, rather than the balance beam, but the principle remains the same. An adjustment of the testing field within a single study species may also make individual differences more pronounced. For example, if the test subject had longer to commit to their initial choice, perhaps because they had further to travel before the Change location was revealed, then they may take longer to adjust their trajectory. This would permit an exploration of how inhibitory control may be mediated by prior investment and effort.

The delay in shifting motion from one previously rewarded target to a new one is regarded as indicative of inhibitory control (Verbruggen and McLaren 2017). Suppressing a previous action and reassigning movement to hit a new target takes time and is indicated by both increased temporal and spatial delays. However, cognitive strategies other than inhibitory control may have influenced performance. One alternative explanation for our findings that does not rely on variation in inhibitory control is that individuals differed in their attention to the task. This could drive differences in their speed of response or indeed whether they responded at all to the apparatus. Likewise, individuals may differ in their visual acuity to such a degree that some simply did not see, or take longer to notice, the reward in the novel location. We did not measure individual attention levels or visual acuity. The rewards that we used were dark mealworms presented on a white Perspex base. Even at a distance (~70 cm) and with low acuity, the contrast should be sufficient to indicate the presence or absence of the worms on the base, and therefore, we suspect that a difference in visual acuity is not an important explanatory factor in our results. A second explanation is that individuals may have chosen which target location to approach prior to the start of a trial, and thus, no reactive inhibitory control was involved. For example, the pheasants may simply repeat a previously rewarded response and thus return to the most recently rewarded food location. Alternatively, individuals may ‘hedge their bets’ and choose to delay targeting one particular reward site until they are much closer to it. In this case, the birds might proceed into the arena on a straight line and only show a distinct bias towards one food location once they have passed the middle of the arena. However, Fig. 4 shows that the pheasants distinctly approached the Go location at the start of a trial, regardless of whether the most recent previous trial had been a Change trial (as was the case for trial 4) or a Go trial (which was the case for trials 5 and 6). Despite the significant initial bias for the Go location, the pheasants were able to adjust their trajectories on the Change trials before arriving at the incorrect food location, which is indicative of inhibitory control. A third alternative explanation is that the pheasants, after experiencing a change in trial 3, learned that when there was a cover attached to the see-saw beam on the side of the Go location the reward displayed at that location will not be accessible. The increased latency on trial 6 could not reflect the engagement of an inhibition process but the fact that the presence of the second cover startled or confused the birds, and led them to proceed with higher caution. However, given the trajectories, and the observation that longer latencies correlated with the birds proceeding further towards the Go location (as measured in the increased distance from the IR crossing point towards the Go location), this possibility seems unlikely. Consequently, it would be helpful, in future, to determine whether an individual’s performance on this putative assay of inhibitory control matches their performance on other tasks considered to be indicative of this cognitive process.

Our interpretation of the results must be tempered by the fact that only 56 of the 194 pheasants tested produced useable trajectory data in all of the six consecutive trials. Failure to participate in any one trial may have been due to altered motivational state or specific stochastic disturbance events (door banging, experimenter coughing, etc.). In such cases, we may expect no bias in our sample population, with such disturbance influencing participation randomly. Alternatively, some birds may have systematically failed to participate. We had instances of some pheasants completing the task in the first 2–4 trials only to cease participating in later trials, whilst other pheasants initially failed to participate, perhaps through fear, but completed later trials consistently. This more systematic failure to participate could have led to a bias in our sample population, with participation influenced by particular individual features. In previous work, we have demonstrated that participation by individual pheasants in a battery of cognitive tasks is influenced by personality, sex, body condition and experience (van Horik et al. 2017). As some of these factors also correspond to inter-individual differences in performance in the Stop-Change task, interpreting their effects becomes confounded.

Pheasants exhibited great variability in our Stop-Change task. Consequently, we believe that our novel paradigm may capture individual differences in control processes as subjects inhibit pre-existing spatial targets and correct their approach towards a changing target location. The task can be applied to a large population within a short period of time, although the demands of repeated engagement over six consecutive trials meant that many (70%) of the original population did not contribute full data in our system. For those pheasants which did participate fully, the test paradigm allows investigation of variation in individual test performance, independent of our measures of motivation. Instead, it appears that an individual’s sex and their body mass may influence their performance in the task. These may be indicative of differences in exertion of inhibitory control or it may arise from physical constraints on task performance. This task offers an important addition to the test battery used to explore the nature of inter-individual differences in inhibitory control and permits direct comparisons within and between species via tests using a common paradigm relying on the deflection of trajectory from a previously rewarded location to a novel reward location only detected after the initial movement had started.

## Electronic supplementary material

Below is the link to the electronic supplementary material.

Supplementary material 1 (WMV 1810 kb)

Supplementary material 2 (WMV 6255 kb)
